# Unusual development of the celiac trunk and its clinical significance

**DOI:** 10.1590/1677-5449.200032

**Published:** 2021-03-15

**Authors:** Serghei Covantev, Natalia Mazuruc, Irina Drangoi, Olga Belic

**Affiliations:** 1 State University of Medicine and Pharmacy “Nicolae Testemitanu”, Laboratory of Allergology and Clinical Immunology, Chişinau, Republic of Moldova.; 2 State University of Medicine and Pharmacy “Nicolae Testemitanu”, Department of Human Anatomy, Chişinau, Republic of Moldova.

**Keywords:** celiac trunk, accessory left hepatic artery, accessory right hepatic artery, additional superior pancreatoduodenal artery, tronco celíaco, artéria hepática esquerda acessória, artéria hepática direita acessória, artéria pancreaticoduodenal superior acessória

## Abstract

We describe a case of unusual development of the celiac trunk observed in the cadaver of 1-year old male child. The celiac trunk branched into five vessels: the splenic, common hepatic and left gastric arteries, the left inferior diaphragmatic artery, and a short trunk that branched into the right inferior diaphragmatic artery and right accessory hepatic artery. Additionally, the manner of branching of the vessel was unusual: it was possible to distinguish two branching points that corresponded to its s-shaped trajectory. There were also other variations of vascular supply, such as the presence of a left accessory hepatic artery, an additional superior pancreatoduodenal artery, and others. It should be noted that multiple developmental variations can be common in clinical practice and clinicians should be aware of them during diagnostic and interventional procedures.

## Resumo

Apresentamos um relato de caso de desenvolvimento incomum do tronco celíaco em um cadáver do sexo masculino de 1 ano de idade. O tronco celíaco ramificou-se para cinco vasos: as artérias esplênica, hepática comum e gástrica esquerda, a artéria diafragmática inferior esquerda e um tronco pequeno que se ramificou para a artéria diafragmática inferior direita e para a artéria hepática direita acessória. Além disso, a forma como o vaso se ramificou foi incomum: é possível distinguir dois pontos de ramificação que correspondem à trajetória em formato de S. Também houve outras variações do suprimento vascular, como a presença da artéria hepática esquerda acessória, da artéria pancreaticoduodenal superior acessória e outras. Cabe observar que a variação de desenvolvimento múltipla pode ser comum na prática clínica, e os médicos devem estar cientes dela durante os procedimentos de diagnóstico e intervenção.

## INTRODUCTION

The celiac trunk is the first major branch of the abdominal aorta. It originates from the ventral aspect of the aorta at the level of the T12-L1 vertebrae. It was originally described as an artery which trifurcates into the common hepatic artery, left gastric artery, and splenic artery. Although this is true, a “classical” trifurcation is seen in 7.1-74.0% of cases depending on the method and studied population.^1^^,^ 2 Such a wide data distribution underlines the importance of complex analysis of anatomical variations, taking into account such factors as sex, age, and population. It also underlines the importance of detailed knowledge of topographical and morphological variability of the vascular supply and collateral blood supply for surgical procedures.^3^ Furthermore, some scientific data is hard to use in medical practice, particularly when populations with different ethnicities are considered.^4^

Anatomical variations of the celiac trunk were classified for the first time by Adachi^5^ in 1928 into 6 types: hepatogastrosplenic, hepatosplenic, gastrosplenic, celiacomesenteric, hepatosplenomesenteric, and hepatomesenteric trunks. Morita proposed an alternative classification and described the celiac trunk and celiacomesenteric variants separately, suggesting 5 types and 15 forms.^6^ Nevertheless, since then a number of classifications have been described in the literature and several authors have proposed their own classifications. Of course, not all variations have been observed until now or are included in these classifications. Moreover, the existence of diverse nomenclatures is complicated for both clinical practice and research.^7^

Detailed knowledge about the anatomy of the celiac trunk is essential when operating on the upper organs of the abdominal cavity. Celiac trunk variations increase the difficulty and the risk of several surgical procedures, particularly laparoscopic cholecystectomy, liver resections, gastric resections, transplant surgery, and others.^8^^,^ 9 Anatomic variations are crucial for surgeons and interventional radiologists; in relation to preoperative computed tomography, MRI, and intraoperative angiography.^10^ We present an anatomical case report of unusual branching of the celiac trunk into five arteries and review its clinical importance.

## CASE DESCRIPTION

**Figure 1:**
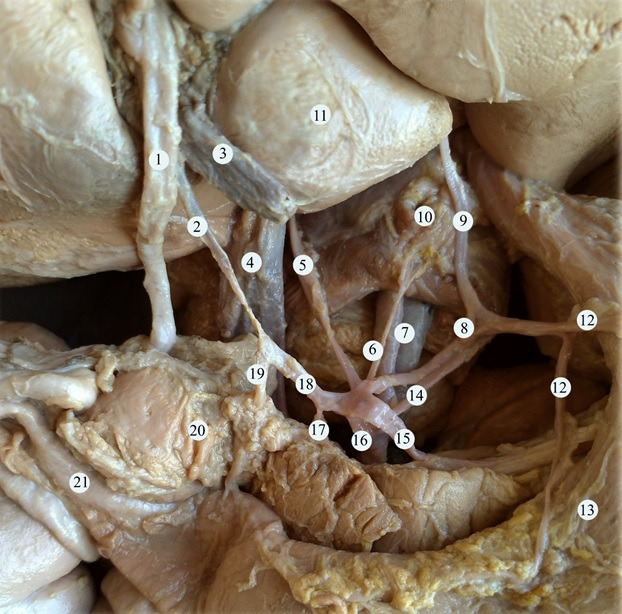
Anatomy of the celiac trunk. (1) common bile duct; (2) proper hepatic artery; (3) portal vein; (4) inferior vena cava; (5) accessory right hepatic artery; (6) right inferior diaphragmatic artery; (7) abdominal aorta; (8) left gastric artery; (9) left accessory liver artery; (10) the diaphragm; (11) the caudate lobe of the liver; (12) arteries of the lesser curvature of the stomach; (13) stomach; (14) the inferior diaphragmatic artery; (15) splenic artery; (16) celiac trunk; (17) additional superior pancreatoduodenal artery; (18) common hepatic artery; (19) gastroduodenal artery; (20) pancreas; (21) duodenum.

**Figure 2:**
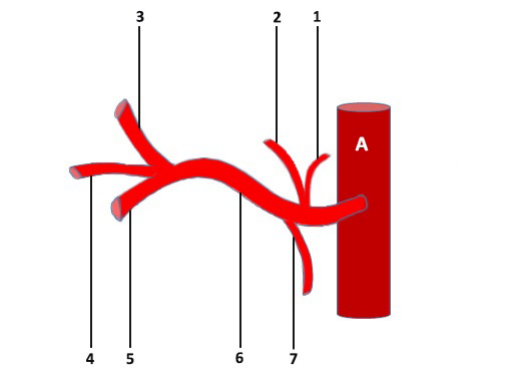
Schematic representation of the celiac trunk. (1) right inferior diaphragmatic artery; (2) right accessory hepatic artery; (3) common hepatic artery; (4) left gastric artery; (5) splenic artery; (6) celiac trunk; (7) left inferior diaphragmatic artery. A = aorta.

During macro and microscopic dissection of the organ complex of the upper floor of the abdominal cavity of a one-year-old child, we discovered an unusual branching pattern of the celiac trunk. This vessel had a sinusoidal (or s-shaped) trajectory and a length of 1.8 cm. The celiac trunk branched into five vessels: the splenic, common hepatic, and left gastric arteries, the left inferior diaphragmatic artery, and a short trunk that branched into the right inferior diaphragmatic artery and right accessory hepatic artery (Figures 1 and 2).

**Figure 3:**
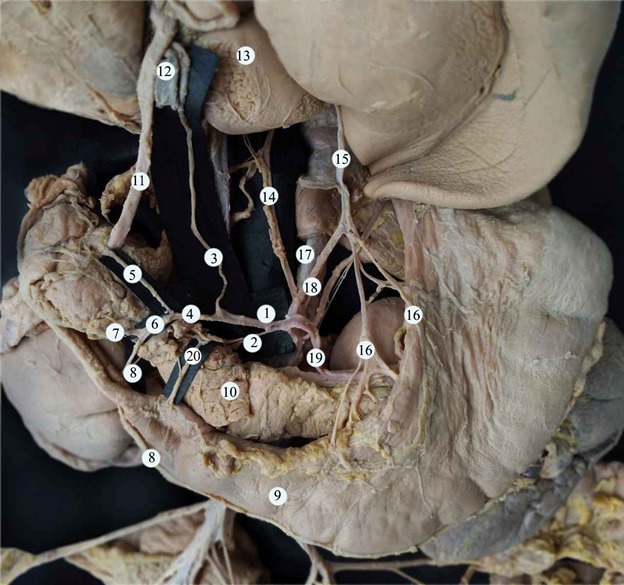
Vascular supply to the organs of the upper abdominal cavity. (1) common hepatic artery; (2) additional superior pancreatoduodenal artery; (3) proper hepatic artery; (4) gastroduodenal artery; (5) superior pancreatoduodenal artery; (6) common trunk; (7) the inferior artery of the pancreatic head; (8) right gastro-epiploic artery; (9) the stomach; (10) pancreas; (11) common bile duct; (12) portal vein; (13) the liver; (14) right accessory hepatic artery; (15) left accessory hepatic artery; (16) arteries to the lesser curvature of the stomach; (17) abdominal aorta; (18) left gastric artery; (19) splenic artery; (20) branch to the wall of the duodenal ampulla.

Additionally, the manner of branching of the vessel was also unusual: it was possible to distinguish two branching points that correspond to its s-shaped trajectory. The first branching point was 6 mm from its point of origin from the aorta. The left inferior phrenic artery and the short trunk originated at this level. The hepatophrenic trunk branched into the right inferior diaphragmatic artery and the right accessory hepatic artery, which entered the hilus of the liver to the left of the inferior vena cava. The second branching point was the division of the vessel into its final branches: the splenic, common hepatic, and left gastric arteries. The left gastric artery branched into four vessels, three of which were directed towards the lesser curvature of the stomach and the left accessory hepatic artery, which entered the parenchyma of the liver close to the venous ligament (Figure 3).

After branching from the celiac trunk, the common hepatic artery gave off an additional superior pancreatoduodenal artery and later divided into the proper hepatic artery and the gastroduodenal artery.

**Figure 4:**
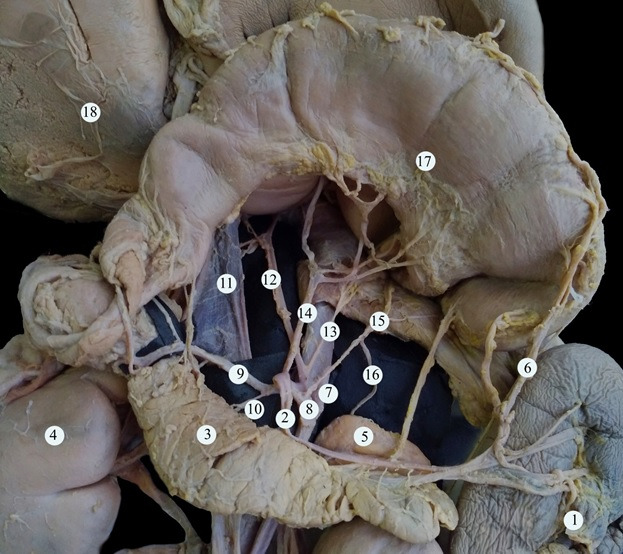
The branches of the celiac trunk (stomach raised up). (1) spleen; (2) splenic artery; (3) pancreas; (4) right kidney; (5) left adrenal gland; (6) the left gastroepiploic artery; (7) abdominal aorta; (8) celiac trunk; (9) common hepatic artery; (10) additional superior pancreatoduodenal artery; (11) inferior vena cava; (12) left accessory hepatic artery; (13) right inferior diaphragmatic artery; (14) gastric left artery; (15) left inferior diaphragmatic artery; (16) superior adrenal artery; (17) the stomach; (18) the liver.

The additional superior pancreatoduodenal artery within the proximity of the pancreatic parenchyma divided into three branches to the pancreatic head and also gave off a branch that supplied the wall of the duodenal ampulla (Figure 4).

**Figure 5:**
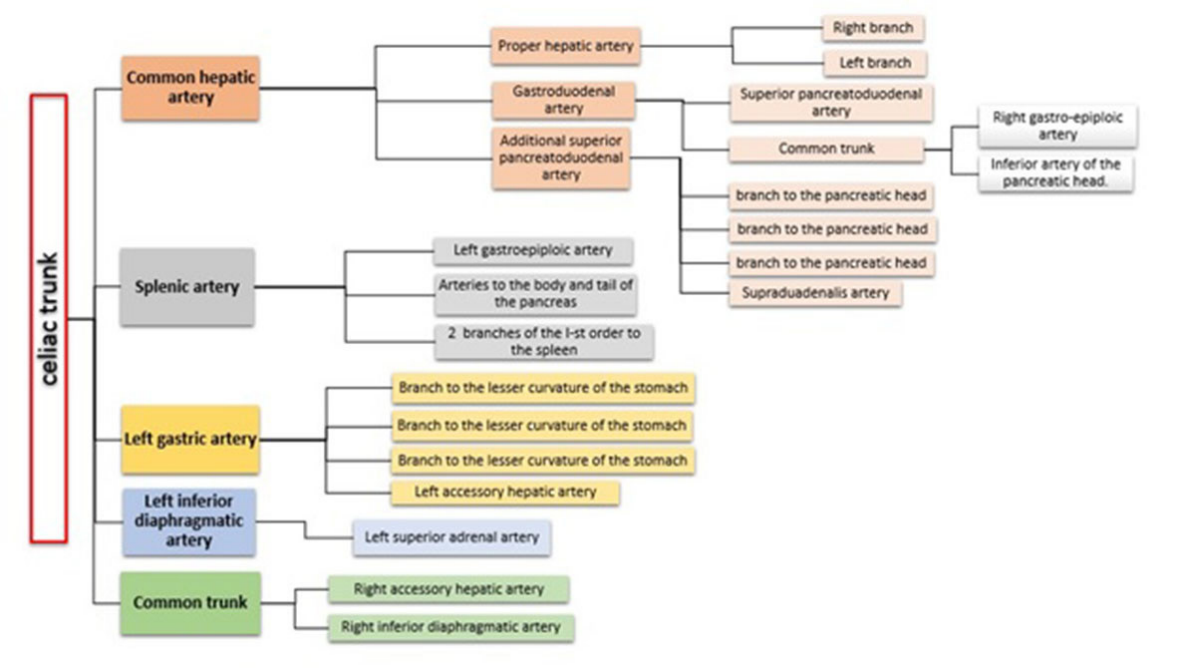
Schematic representation of vascular supply from the celiac trunk.

The gastroduodenal artery branched into the superior pancreatoduodenal artery and a common trunk, which in turn branched into the right gastro-epiploic artery and lower artery of the pancreatic head. The left gastro-epiploic artery branched from the splenic artery (Figure 3). A schematic representation of vascular supply from the celiac trunk is presented in Figure 5.

## DISCUSSIONS

The celiac trunk and the superior mesenteric artery develop from the 10th and 13th metameric ventral splanchnic (vitelline) arteries that supply the embryonic gut. Initially the vitelline arteries are connected by a ventral longitudinal anastomosis. After the longitudinal anastomosis between the 12th and 13th vitelline arteries has disappeared, the superior mesenteric artery develops from the 13th vitelline artery. Anomalies of the celiac trunk and superior mesenteric artery can occur due to persistence of the ventral longitudinal anastomosis and regression of some of the roots of vitelline arteries.^7^

A large retrospective evaluation of 5002 patients who underwent spiral CT and digital subtraction angiography demonstrated celiac trunk variations in 9.64% of cases.^11^ In cadaveric studies, the celiac trunk branched into a common hepatic artery, a left gastric artery, and a splenic artery in 43.6% of dissections. A true tripod was seen in 7.1% and a false tripod in 36.4%. Additional branches were frequent and can be encountered in 47.9%. Celiac trunk tetrafurcation was observed in 12.9%, pentafurcation in 12.9%, hexafurcation in 1.4%, and heptafurcation in 0.7%.^1^ Our present case represents a pentafurcation of the celiac trunk although the difference is that it is not a true pentafurcation. The celiac trunk had an initial division into three arteries (left inferior phrenic artery; a common trunk for the right inferior phrenic artery and accessory right hepatic artery; and a hepatogastrosplenic trunk).

One of the most common additional branches of the celiac trunk is to the phrenic arteries. The phrenic arteries originate from the celiac trunk as a single phrenic in 12.6% of cases. The right phrenic artery originates directly from the celiac trunk in 3.85-30.7% and the left in 23.07-40.3%.^12^^,^ 13 Although the phrenic arteries commonly begin from the celiac trunk, this case is unusual because of the fact that we had a common trunk between the accessory right hepatic artery and the right inferior phrenic artery. The left inferior phrenic artery began as a separate trunk from the celiac trunk. Knowing the origin of the inferior phrenic arteries is important in a number of clinical situations. For example, transcatheter arterial chemoembolization (TACE) treatment in the unresectable hepatocellular carcinoma.^14^ Hepatic neoplasms are commonly supplied by the hepatic arteries, but extrahepatic collateral vessels can also supply an entire or at least part of a hepatic neoplasm. Inferior phrenic arteries can often be an important source of collateral supply to hepatocellular carcinoma.^15^^,^ 16

Massive hemoptysis due to inferior phrenic arteries may also be encountered in patients with chronic pancreatitis, trauma or surgery, and bleeding caused by gastroesophageal problems (Mallory-Weiss tear or gastroesophageal cancer).^15^^,^ 17 Diseases of the lungs associated with fibrosis and decreased pulmonary blood flow, such as bronchiectasis, cystic fibrosis, tuberculosis, sarcoidosis, chronic pneumonia, or congenital pulmonary artery stenosis may cause development of a transpleural systemic-pulmonary artery anastomosis. If one of the aforementioned lung abnormalities is located at the base of the lung, the inferior phrenic artery can also be the source of massive hemoptysis.^15^

Michel classified the vascular supply of the liver into 10 types and Hiatt later modified this classification.^18^^,^ 19 Nevertheless, anatomical variations of the vascular supply of the liver often cannot be classified.^20^ In such cases, they must be evaluated individually, taking into account the morphological particularities and their clinical significance. Abnormal vascular supply to the liver can be seen in at least 20% of cases.^21^ An accessory right hepatic artery that originates from the celiac trunk is seen in 5.16% cases.^22^ A left accessory hepatic artery that originates from the left gastric artery is seen in 0.3% of cases,^23^ although other authors have reported a higher incidence of this variation (19.86%).^24^ In our case, there was an accessory right hepatic artery, which began from the celiac trunk as a common trunk with the right inferior diaphragmatic artery. The accessory left hepatic artery began from the left gastric artery. According to Michel’s classification, the presence of both right and left accessory hepatic arteries is a type VII variation and is seen in 0.2% of cases.^21^

The importance of these anatomic variations has its relevance in TACE, transarterial radionuclide therapy (TART), and placement of infusion pumps. When placed surgically, these catheters are typically placed in the gastroduodenal artery in a retrograde direction with the catheter tip in the proximal gastroduodenal artery or in the proper hepatic artery. The gastroduodenal artery distal to the catheter insertion site and branches supplying blood to the stomach and duodenum are ligated to prevent nontarget infusion. When they are placed percutaneously, catheters are typically positioned in the proper hepatic artery to infuse both hepatic arteries. In cases with variant anatomy, an alternate catheter position or more than one catheter must be considered preoperatively to ensure adequate tumor perfusion.^25^ In our case, there were two main hepatic arteries and two accessories, which would have constituted a complicated case for this procedure.

The superior pancreatoduodenal artery can have variable origin, number, and course. Several authors report an accessory artery in 10.7-20% of cases, but the majority of researchers report that it is rarely double.^26^ In our case, the additional superior pancreatoduodenal artery branched into four arteries, three of which vascularized the parenchyma of the pancreas, while the fourth vessel supplied the duodenum.

Although this is a single case report of an unusual vascular anatomy of the abdomen, it demonstrates the complexity of human anatomy and has several important clinical implications for the day-to-day practice of surgeons, interventional radiologists, and other specialists.

## CONCLUSIONS

During macroscopic dissection, we identified a celiac trunk that branched into five vessels: the splenic, common hepatic, and left gastric arteries, the left inferior diaphragmatic artery, and a short trunk that branched into the right inferior diaphragmatic artery and accessory right hepatic artery. Textbook anatomy gives an overview of the most common types of developmental variation. Nevertheless, in reality, anatomy is often more complicated. Several surgical procedures depend on good knowledge of the regional anatomy, particularly laparoscopic cholecystectomy, liver resections, gastric resections, transplant surgery, and others. Anatomic variations are crucial for surgeons and interventional radiologists. Vascular variations are also important in TACE and TART procedures or placement of infusion pumps. This case demonstrates that there is much that can be learned from cadaver dissections even in the era of advanced imaging techniques.
